# Comparison of clinical outcomes with and without volar lunate facet fragments in dorsal displaced distal radius fractures

**DOI:** 10.1051/sicotj/2020047

**Published:** 2021-01-08

**Authors:** Hiroyuki Obata, Kiyohito Naito, Yoichi Sugiyama, Nana Nagura, Kenji Goto, Ayaka Kaneko, So Kawakita, Kazuo Kaneko

**Affiliations:** 1 Department of Orthopaedics, Juntendo University School of Medicine 2-1-1 Hongo Bunkyo-ku 113-8421 Tokyo Japan

**Keywords:** Distal radius fractures, Volar lunate facet fragments, Dorsal displaced fractures, Proximal volar locking plates

## Abstract

*Introduction*: Although many clinical studies about distal radius fracture (DRF) accompanied by volar lunate facet fragments (VLFF) have recently been reported, none focus on the direction of displacement of distal fragments. Many previous cases with difficulty in treating DRF with VLFF were volar-displaced fractures. Thus, the postoperative risk for re-displacement is different between volar- and dorsal-displaced fractures with VLFF. The aim of this study is to compare the outcome of dorsal-displaced fractures treated using proximal volar locking plates (PVLP) between those with VLFF and those without, in order to reconsider the indications of distal volar locking plates (DVLP) and investigate the possibility of treating dorsal-displaced DRF with VLFF using PVLP. *Methods*: The subjects were 122 patients with dorsal-displaced DRFs treated using PVLP (42 males and 80 females, mean age: 59.2 years old). The patients were divided into 13 patients with VLFF group and 109 patients without VLFF group, and the clinical outcomes at 12 months after surgery were compared. *Results*: No significant difference was noted on any evaluation between the groups. In addition, no postoperative re-displacement of VLFF was observed and bone union was confirmed. Furthermore, no osteoarthritic change was noted in all patients. *Conclusions*: We confirmed that surgical treatment for dorsal-displaced DRF using PVLP is possible even in cases of DRF with VLFF. In addition, DVLP is an implant with a high complication risk; therefore, it may be necessary to reconsider the use of DVLP for dorsal-displaced DRF with VLFF treatable by PVLP.

## Introduction

Many studies on the treatment strategy for distal radius fracture (DRF) with volar lunate facet fragments (VLFF) have recently been reported [1–4]. In 2014, Beck et al. reported that a 15-mm or smaller longitudinal diameter of VLFF in the lateral view on plain radiography is a risk factor for postoperative re-displacement [5], and after this report, many treatments strategies based on the size of VLFF have been discussed. However, volar locking plates are often selected based on the VLFF size and to our knowledge, there has not been reported that importance to the displacement direction of distal bone fragments. Indeed, distal volar locking plates (DVLP) are frequently used because of the small size of VLFF [6, 7]. However, many complications of DVLP, such as flexor tendon injury and crushing of VLFF, have been reported, demonstrating them to be an implant with a high risk for postoperative complications [8–11]. Therefore, reconsideration of their indications may be necessary.

Among DRFs with VLFF, many with difficulty in treatment due to postoperative re-displacement are of volar-displaced fractures [12–14]. Our algorithm for treating distal radius fractures uses proximal volar locking plates (PVLP) for dorsal displaced fractures and DVLP for volar displaced fractures [15]. Therefore, in this study, based on the hypothesis that PVLP can be used to treat dorsally displaced fractures even with VLFF, the clinical outcomes of PVLP fixation for dorsally displaced fractures with and without VLFF were compared. The purpose of this study is to prevent unnecessary use of DVLP, which has many postoperative complications, just because “VLFF is small”.

## Materials and methods

### Patients

This study was approved by the ethics committee for medical research of our university (No. 17-250), and informed consent was received from all patients.

The subjects were 122 patients with dorsal-displaced DRFs treated by reduction and fixation using PVLP (42 males and 80 females, mean age: 59.2 years old) between January 2012 and December 2018. In the distal fragments of the distal radius fractures, the free bone fragments in the volar lunate facet fragment in which the longitudinal diameter of the volar bone cortex is 10 mm or less are defined as the volar lunate facet fragment [16]. The patients were divided into 13 patients with VLFF (VLFF (+) group: 9 males and 4 females, mean age: 60.0 years old) and 109 patients without VLFF (VLFF (−) group: 33 males and 76 females, mean age: 59.2 years old), and the clinical outcomes at 12 months after surgery were compared. The range of motion of the wrist, visual analog scale (VAS), quick disabilities of the arm, shoulder and hand (Q-DASH) score, and Mayo wrist score were investigated as evaluation items. The fracture type according to the AO classification on preoperative plain X-ray images was C1 in 4 patients, C2 in 2, and C3 in 7 in the VLFF (+) group, and A2 in 32, A3 in 5, C1 in 65, C2 in 5, and C3 in 2 in the VLFF (−) group. PVLP was used in all patients regardless of VLFF.

1. VA-TCP 2.4^®^ (Depuy Synthes, Tokyo, Japan) for 68 patients.
2. Acu-Loc 2 proximal plate^®^ (Nihon Medical Next, Osaka, Japan) for 23.
3. DVR^®^ (Zimmer Boimet, Tokyo, Japan) for 21.
4. Dual-Loc V17^®^ (MEIRA, Aichi, Japan) for 9.
5. Stellar 2^®^ (HOYA, Tokyo, Japan) for one.

The selection of VLP depended on the timing of the surgery, not the plate design. VLP fixation was applied through the trans-FCR approach.

### Surgical technique

**Figure 1:**
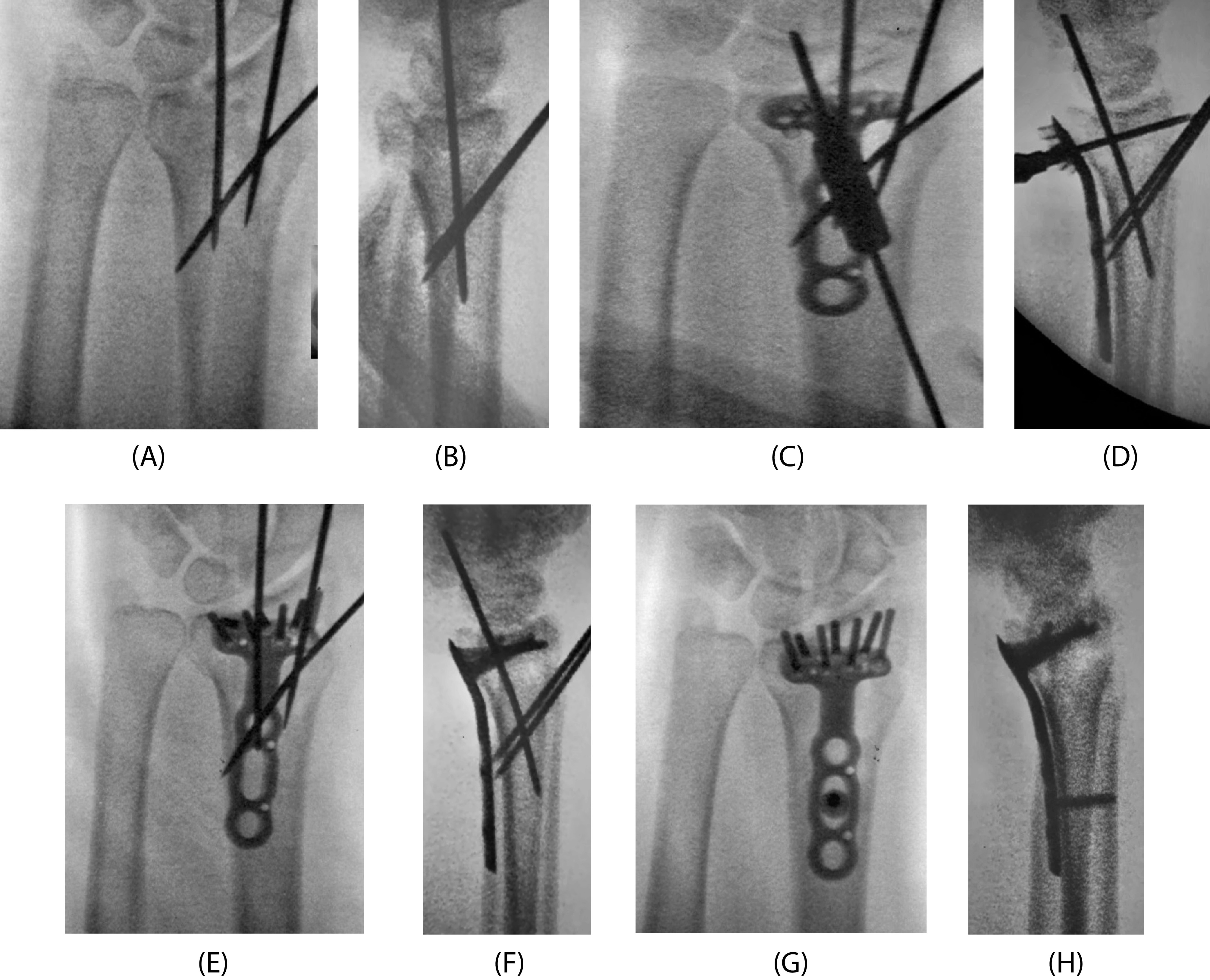
Surgical procedure for distal radius fractures with dorsal-displaced VLFF. From the dorsal side of the fracture region, 1.8-mm Kirschner wire was inserted and dorsal displacement of distal bone fragments was reduced using the Kapandji technique ((A) PA view, (B) Lateral view). Dorsal volar bone fragments were pressed to each other to be bound to reduce the gap in the fracture region ((C) PA view, (D) Lateral view). The plate was placed at a position at which distal locking screws support the dorsal side of the radial joint surface ((E) PA view, (F) Lateral view). The distal bone fragment was reduced and fixed according to the condylar stabilizing method ((G) PA view, (H) Lateral view).

**Figure 2:**
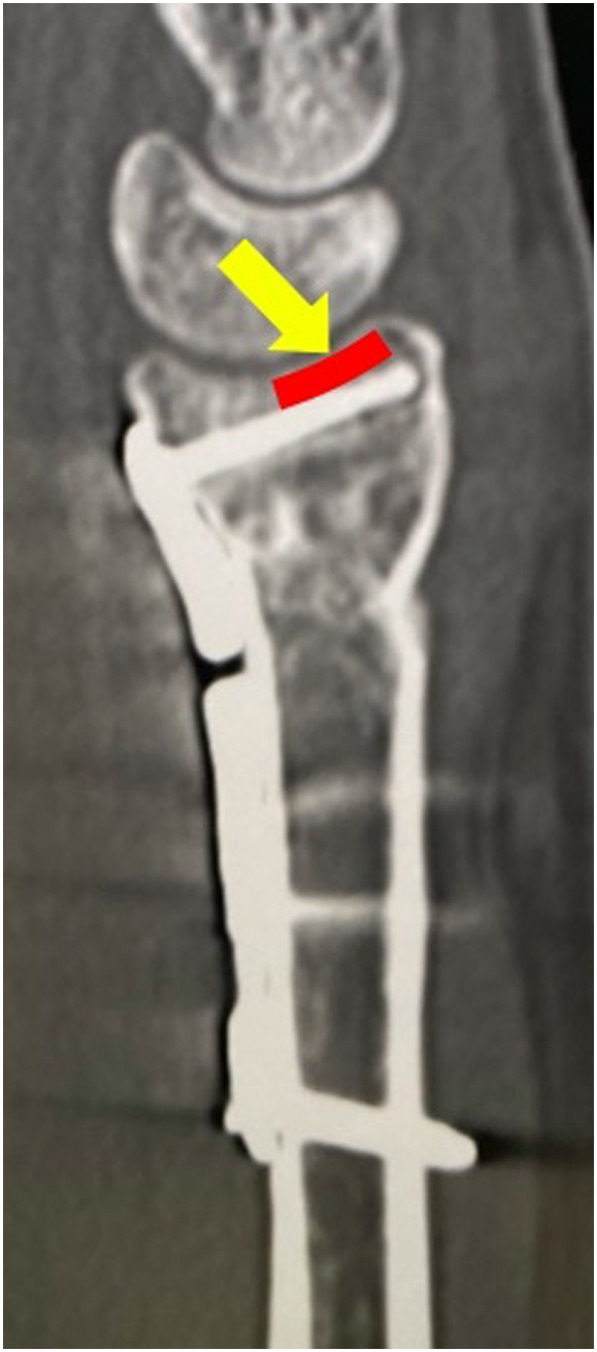
The theory of osteosynthesis and surgical technique of PVLP. In order to prevent dorsal re-displacement, it will be more important to obtain good subchondral support of the dorsal side of the radial facet with PVLP compared with buttress effect by covering the volar lunate facet fragment (*yellow arrow*: the dislocation direction of the carpal, *red line*: the dorsal side of the radial facet supported by the locking screw).

Zenke et al have classified it as “intramedullary” when the volar cortex of the distal bone fragment was invaginated medially to the proximal part, “anatomical” when the volar cortex met the volar cortex, and “extramedullary” when the volar cortex of the distal bone fragment was located laterally to the proximal part [17]. In surgery, 1.8-mm Kirschner wire was first inserted from the dorsal side of the fracture region, and dorsal displacement of distal bone fragments was reduced from “intramedullary” to “anatomical” or “extramedullary” using the Kapandji technique (Figures 1A and 1B). This was targeted by placing the plate to prevent it from lifting from distal bone fragments. Then, in the VLFF (+) group, an elevator was inserted into the intramedullary canal from the volar side of the fracture region when there was an intramedullary depressed bone fragment, and a reduction was applied by lifting the depressed bone fragment from the intramedullary canal in order to press it to the lunate bone. In addition, the volar fragment was pushed to the dorsal fragment over the plate to reduce the gap in the fracture site (Figures 1C and 1D). For the plate, PVLP was selected because the distal screw is inserted on the dorsal side of the radial facet to support the dorsal-displaced bone fragment displaced toward the dorsal. The plate placement site was set at a level at which the plate placement site did not cross the watershed line and the dorsal side of the radial joint surface was able to be supported by inserting distal locking screws (Figures 1E, 1F, and 2). The volar radius also presents a concave profile in the sagittal plane (the pronator fossa). This feature is limited distally by a ridge called the watershed line and allows the application of implants of substantial profile [18]. After distal locking screws insertion, shortening of the fracture region and reduction of ulnar displacement of proximal bone fragments were performed according to the Condylar stabilizing technique [19], followed by fixation by proximal cortical screw insertion (Figures 1G and 1H). Immobilization after surgery was not necessary, and movement of the wrist joint and fingers permitted early after surgery.

### Statistical analysis

Data are presented as the mean ± standard deviation (SD) and were analyzed for significant differences by the Mann–Whitney *U* test (Prism 4, GraphPad Software, San Diego, CA). Differences were considered significant at *P* < 0.05.

## Results

On evaluation at 12 months after surgery, in the VLFF (+) group, the range of motion of the wrist joint was 72.6 ± 6.9° flexion and 69.2 ± 8.5° extension, and forearm pronation and supination were 83.0 ± 6.9° and 84.6 ± 7.1°, respectively. The VAS, Q-DASH score, and Mayo wrist score were 1.3 ± 1.2/10, 10.3 ± 7.1/100, and 93.1 ± 4.8/100, respectively. In the VLFF (−) group, the range of motion of the wrist joint was 73.3 ± 10.7° flexion and 73.6 ± 10.7° extension, and forearm pronation and supination were 83.6 ± 7.7° and 85.2 ± 7.2°, respectively. The VAS, Q-DASH score, and Mayo wrist score were 0.9 ± 1.0/10, 9.6 ± 10.0/100, and 93.5 ± 7.0/100, respectively. There were no major perioperative complications in either group. No significant difference was noted in any evaluation between the groups (Table 1). In addition, no postoperative re-displacement of VLFF was observed and the bone union was confirmed. Furthermore, no osteoarthritic change was noted in all patients.

**Table 1:** Comparison of clinical outcomes at 12 months after surgery between VLFF (+) and VLFF (−) groups.

	VLFF (+)	VLFF (−)	*P*-value
Cases	13	109	N.S.
Wrist F/E (°)	72.6/69.2	73.3/73.6	N.S.
Forearm P/S (°)	83.0/84.6	83.6/85.2	N.S.
VAS	1.3	0.9	N.S.
Q-DASH score	10.3	9.6	N.S.
Mayo score	93.1	93.5	N.S.

VLFF: volar lunate facet fragment, F: flexion, E: extension, P: pronation, S: supination, VAS: visual analog scale, Q-DASH: quick disabilities of the arm, shoulder, and hand, N.S.: no significant difference.

## Discussion

The treatment strategy for DRF with VLFF focuses only on the size of VLFF and the displacement direction of fractures is not taken into consideration in many cases. However, many cases that were difficult to treat due to postoperative re-displacement were of volar-displaced DRF with VLFF, and re-displacement on the volar side developed after surgery because PVLP was selected for these volar-displaced fractures or DVLP was selected, but the plate placement site was inappropriate [12–14]. Based on these reports, we cover VLFF and sufficiently apply buttress fixation as a treatment strategy using DVLP, considering that volar-displaced DRF with VLFF has a high risk for postoperative displacement [15]. In 2004, Harness et al. reported that treatment of DRF with VLFF using an existing volar locking plate is difficult due to its anatomical characteristics [20]. However, new DVLP applicable to volar-displaced fractures distal to the watershed line have recently been developed, and treatment of volar-displaced DRF with VLFF using DVLP has improved [15, 21].

On the other hand, in dorsal-displaced fractures with VLFF, we consider secondary displacement to be caused by pulling by the short radiolunate ligament attached to VLFF through the dorsal displacement of carpal VLFF. Therefore, to prevent postoperative re-displacement of VLFF, prevention of carpal dorsal displacement is necessary, for which support of the dorsal side of the radial joint surface is important [22].

Accordingly, the selection of PVLP in which distal locking screws support the dorsal side of the radial joint surface is rational. Cases of postoperative volar re-displacement of dorsal-displaced DRF with VLFF have been occasionally reported, but most may have been due to excess reconstruction of volar tilt [23, 24]. Orbay et al. treated patients with postoperative volar re-displacement of dorsal-displaced DRF with VLFF by open wedge osteotomy and achieved favorable postoperative outcomes by reducing volar tilt. They mentioned the importance of reducing the load on VLFF even by allowing slight dorsal volar tilt. They also stated that excess reconstruction of volar tilt is a risk factor for volar re-displacement [23]. Thus, we considered that the risk for postoperative re-displacement can be prevented by avoiding loading on VLFF by not excessively moving the load axis toward the volar side through avoiding excess reconstruction of volar tilt. In this study, the mean postoperative volar tilt was 8.5 (2–15) due to the use of PVLP in the VLFF (+) group and no over-correction-induced volar re-displacement developed after surgery in any patient.

When DVLP are selected for dorsal-displaced DRF with VLFF, the carpal bone may be re-displaced toward the dorsal side after surgery because the distal locking screws mainly support the center of the radial joint surface and VLFF may be secondarily displaced by being pulled by the short radiolunate ligament. Regarding the relationship between volar tilt and the flexor tendon, Wurtzel et al. pointed out that insufficient reconstruction of volar tilt may increase the risk for postoperative flexor tendon injury [25]. Moreover, the pronator quadratus muscle is damaged in many cases of dorsal-displaced DRF and covering the plate after repairing the pronator quadratus muscle after plate placement is often difficult. Therefore, when DVLP are selected for dorsal-displaced DRF with VLFF, they cannot be covered with the pronator quadratus muscle and volar tilt usually decreases due to re-displacement, increasing the risk for postoperative iatrogenic flexor tendon injury.

Some recent studies stated the importance of fixation of VLFF [26–28], but it may be difficult to fix VLFF even though additional fixation with screws, anchors, or wire is applied because the bone mass and bony tissue are insufficient. It may cause blood circulation disorder, inducing crushing and necrosis of VLFF because the surrounding soft tissue is dissected to try to fix VLFF, and screws and wire are inserted. We fixed dorsal-displaced DRF with VLFF using PVLP in this study. As a dissection of soft tissue attached to VLFF was minimized, there was no crushing or necrosis of VLFF, and the bone union was confirmed in all patients.

In conclusion, the clinical outcomes of dorsal-displaced DRF were compared between patients with and without VLFF. It was comparable regardless of VLFF, clarifying that reduction and fixation of the dorsal-displaced fracture using PVLP are possible even in cases of DRF with VLFF. DVLP is an implant with risks of many complications and their use should be limited to volar-displaced fractures.

## Declarations

### Conflict of interest

The authors declare that they have no conflict of interest.

### Ethics approval

The study was approved by the ethics committee for medical research of our university (No. 17-250).

### Consent to participate

Informed consent was received from all patients.

### Consent for publication

Informed consent was received from all patients.

### Availability of data and material

The datasets during and/or analysed during the current study available from the corresponding author on reasonable request.

### Code availability

The datasets during and/or analysed during the current study available from the corresponding author on reasonable request.

### Authors’ contributions

HO (first author) mainly wrote this manuscript, acquisition of data, analysis and interpretation of data. KN (corresponding author), KG, YS, and KK mainly performed the conception and design of this study. HO, KG, AK, and NN performed acquisition of data, analysis and interpretation of data.
